# ANGPTL4 suppresses the profibrogenic functions of atrial fibroblasts induced by angiotensin II by up-regulating PPARγ

**DOI:** 10.22038/IJBMS.2023.69196.15077

**Published:** 2023

**Authors:** Xi Zhu, Xiaogang Zhang, Wei Gu, Hanjun Zhao, Shuwen Hao, Zhongping Ning

**Affiliations:** 1 Department of Cardiology, Shanghai Pudong New Area Zhoupu Hospital (Shanghai Health Medical College Affiliated Zhoupu Hospital) shanghai 201318, China

**Keywords:** Atrial fibrillation, Atrial fibrosis, Lipoprotein lipase, Peroxisome proliferator-activated receptor-γ, Triglyceride

## Abstract

**Objective(s):**

The present study’s objective was to investigate the association between angiopoietin-like 4 (ANGPTL4) levels and the prognosis of Atrial fibrillation (AF), the causative effect in angiotensin II- (Ang II) induced AF, and its underlying mechanisms.

**Materials and Methods:**

Baseline serum ANGPTL-4 concentrations were measured in 130 patients with AF. Rat atrial fibroblasts were isolated from 14-day-old SD rats and transfected with Ang II treatment. Transfected cells were divided into: The control group, ANGPTL4-OE group, Ang II group, and Ang II+ANGPTL4-OE group. Transfected cells were used to analyze fibroblasts’ proliferation, migration, and collagen production at the cellular level. RT-qPCR and western blotting evaluated the ANGPTL4-targeted gene and PPARγ-Akt pathway.

**Results:**

In patients with AF, serum ANGPTL4 concentrations decreased significantly compared with the healthy group. ANGPTL4 mRNA and protein expressions were significantly down-regulated in Ang II-induced cardiac fibroblasts. ANGPTL4 overexpression potentially attenuated Ang IIinduced fibroblast proliferation, migration, and collagen production in atrial tissue. ANGPTL4 inhibited the signaling proteins, such as PPARγ, α-SMA, and Akt.

**Conclusion:**

Our experimental data speculate that ANGPTL4 is a key factor in regulating AF progression. Therefore, increasing ANGPTL4 expression could be an effective strategy for AF treatment.

## Introduction

Atrial fibrillation (AF) has been a growing concern in recent years. It continues to be the most common arrhythmia encountered in clinical practice and a leading possible cause of stroke and heart attack (1, 2). Accumulating reported evidence has speculated that atrial fibrosis is the main pathological attribute of atrial structure in AF. Atrial fibrosis is closely associated with structural reconstruction and maintenance and is crucial to AF progression (3, 4). Excessive deposition of atrial tissue’s extracellular matrix (ECM) in animals and the biopsies case of heart failure enhanced atrial fibrosis (5). This evidence demonstrates the relationship between atrial fibrosis and AF, although revealing the impact of tissue fibrosis on the incident and persistence of AF remains a critical challenge. In addition, better exploration of the underlying mechanisms of atrial fibrosis is inevitable for developing potential strategies to treat structural remodeling.

Angiopoietin-like 4 (ANGPTL4) is an angiopoietin family member and a hepatic fibrinogen/angiopoietin-related protein (HEARP) (6), peroxisome proliferator-activated receptor-γ (PPARγ) angiopoietin-related gene (PGAR) (7), or fasting-induced adipose factor (FIAF) (8). ANGPTL4 is a secretory protein observed in the liver, placenta, adipose tissue, and ischemic tissue (7, 8). Recent studies reported that ANGPTL4 could regulate angiogenesis, tumorigenesis, vascular permeability, glucose metabolism, lipid metabolism, energy homeostasis, wound healing, cell differentiation, redox reaction, and inflammatory response. Several physiological conditions, including fasting, pregnancy, lactation, adipocyte differentiation, and hypoxia could up-regulate ANGPTL4 expression. In addition, plasma ANGPTL4 concentration has been increased in short-term hypothermia, chronic caloric restriction, a deficient calorie diet, free fatty acids, and a high-fat high-energy diet. ANGPTL4 plays a critical role in lipid metabolism by suppressing the lipoprotein lipase (LPL) activity, which is hydrolyzed plasma triglyceride (TG) to free fatty acids (9). Previous studies reported that ANGPTL4 overexpression decreases the activity of LPL, and circulating TG levels increase. 

In the initial research, the risk of AF recurrence was correlated with serum ANGPTL4 levels (10). A lipoprotein lipase inhibitor called ANGPTL4 slows the breakdown of plasma TG into free fatty acids. Fatty acids’ negative feedback on the enzyme ANGPTL4 also controls its activity, maintaining a stable state (11). This evidence suggests that fatty acids, particularly polyunsaturated fatty acids, boost the expression of ANGPTL4 in cardiomyocytes and liver cancer cells (12, 13). Patients with AF exhibit abnormalities in fatty acid metabolism and atrial fatty acid transport, which are hypothesized to be connected to a higher risk of an AF attack following cardiac surgery (14). In addition, patients with AF had higher levels of saturated fatty acids and lower levels of polyunsaturated fatty acids in their plasma, which may indicate that free fatty acids have a role in the onset and development of AF.

The pathogenesis of AF involves inflammation, and when inflammatory mediators move into atrial tissue in excess, they change the structural and electrical characteristics of the tissue (15). Ang II can enhance atrial fibrosis and inflammation, which makes AF more susceptible to being induced (16). Additionally, ANGPTL4 exhibits a strong anti-inflammatory impact. In the myocardial infarction animal model, ANGPTL4 therapy boosted the anti-inflammatory macrophages and significantly enhanced heart function (17). An earlier study found that mice with Ang II and ANGPTL4 had considerably decreased atrial fibrosis compared with mice with Ang II alone and had higher expression of α-SMA, collagen I, and collagen III (18). However, in this study, we evaluated the protective effect of ANGPTL4 in Ang ll-induced AF.

## Materials and Methods

***Study population***

Patients who underwent radiofrequency ablation between July 2016 and June 2018 in the Department of Cardiology, Shanghai University of Medicine & Health Sciences affiliated Zhoupu Hospital, were enrolled in this study (n=130). The exclusion criteria were as follows: nonvalvular AF, chronic pulmonary heart disease, vein thrombosis, pulmonary embolism (PE), aortic dissection (AD), sepsis, severe dysfunction of liver or kidney, surgery or trauma within one month, malignant tumors, and hematologic disorders. The control group included 130 serum samples from healthy subjects who received physical examination with matched age and gender. Written informed consent was obtained from all participants. The Ethics Committee of Shanghai University of Medicine & Health Sciences approved this current study.

***Serum sample collection and assay***

Peripheral blood samples were collected from each fasting patient on the second morning after being hospitalized, and serum was separated and stored at −80 °C after centrifugation (4,000 g, 4 ^°^C, 15 min). The serum concentrations of ANGPTL4 (DY3485, R&D Systems, USA), TGF-β1 (DB100B, R&D Systems, USA), CTX-I (ml028171, Shanghai Enzyme-linked Biotechnology Co., China), PICP (ml057471, Shanghai Enzyme-linked), PIIINP (ml063225, Shanghai Enzyme-linked), and PPARγ (XG-01E7159, Shanghai SiGe Biotechnology Co., Ltd, China) were determined by ELISA.

***Isolation of rat atrial fibroblasts***

The male newborn SD rats (14-day-olds) were anesthetized using pentobarbital sodium (60 mg/kg) and ketamine (50 mg/kg) intraperitoneal injection under sterile conditions before thoracotomy. Then, the heart was isolated aseptically and placed in a petri dish. The atrial tissues were isolated from the heart and washed to remove congestion and excess epicardial tissues. Afterward, atrial tissues were cut into pieces of about 1 mm 3 in size, followed by trypsin digestion and differential centrifugation. The atrial fibroblasts were obtained and infused in Dulbecco’s modified Eagle medium (Gibco, Grand Island, NY, USA) with 10% fetal bovine serum for incubation at 37 ^°^C with 5% CO_2_. The culture medium was replaced every three days, the cell morphology was observed, and pictures were captured under an inverted microscope.

***Cell culture***

Cells were grown and cultured in DMEM containing 10% (vol/vol %)-fetal bovine serum (FBS) in a humidified atmosphere at 37 ^°^C with 5% CO_2_. Cells in passages 2-3 were used in experiments. With different concentrations of Ang II (0.1, 1, 10 μM), cells were treated for 24 hr to reach 70% confluence. GW9662 (10 μM, HY-16578, MedChemExpress) was used to inhibit PPARγ activity for the inhibitor experiment.

***Cell transfection and Ang II treatment***

The rat atrial fibroblasts were transfected, followed by exposure to Ang II for 24 hr. Cells were grouped into Vector group (transfection with empty pcDNA3.1), ANGPTL4-OE group (transfection with pcDNA3.1-ANGPTL4), Ang II group (transfection with empty pcDNA3.1 before exposure to 1 μM Ang II), Ang II+ANGPTL4-OE group (transfection with pcDNA3.1-ANGPTL4 before exposure to 1 μM Ang II). Human ANGPTL4 cDNA (NM-139314.3) were amplified and then cloned into a pcDNA3.1 vector (GenePharma Co. Ltd, Shanghai, China) to construct ANGPTL4 overexpressing plasmids (ANGPTL4-OE). The empty pcDNA3.1 vector served as a negative control. After using the Lipofectamine 2000 reagent (Invitrogen, Carlsbad, CA, USA), transfection was embarked upon. RT-qPCR and western blotting verified the efficiency of ANGPTL4 gene transfection.

***Cell viability assay***

After cell transfection and Ang II treatment, the diluted atrial fibroblast cell (2×10^3^) was seeded into a 96-well plate (100 µl/well). 10 µl of CCK-8 kit (Tokyo, Dojindo, Japan) was added to each plate well and incubated at 37 ^°^C for 4 hr. The absorbance at 450 nm wavelength was measured. Three replicates were used for each sample. The absorbance value of each group was normalized to that of the control group.

***Cell migration assay***

The transfected cells (5×10^4^) were seeded and stimulated with or without Ang II. With serum-free DMEM medium, the indicated cells were grown in the upper chamber for 24 hr. After that, non-migratory cells were removed, and the lower chamber’s membranes containing cells were fixed and stained. Under a microscope, the migratory cells were measured.

***RT-qPCR***

TRizol (Invitrogen, USA) was used to extract total RNA from mice atrial tissues. Reverse transcription was carried out to convert total RNA into complementary DNA (cDNA). mRNA was amplified by Real-Time Quantitative PCR (RT-qPCR) using SYBR Green reagent (TaKaRa, Japan) in an ABI Prism 7700 Real-Time PCR system (Applied Biosystems, USA). The list of primer sequences used was as follows: ANGPTL4 (forward: 5′-AGA AGT TGG AGA TGC AGA GGG AC-3′; reverse: 5′-CCA CAA GAG CAC CAT TGA GTG TAT-3′); COL1A1 (forward: 5′-GCC TCA GCC ACC TCA AGA GA-3′; reverse: 5′-GGC TGC GGA TGT TCT CAA TC-3′); COL3A1 (forward: 5′-CGG GCA AGA ATG GAG CAA AG-3′; reverse: 5′-ACC AGG GAA ACC CAT GAC AC-3′); PPARγ (forward: 5′-ATT CTG GCC CAC CAA CTT CGG-3′; reverse: 5′-TGG AAG CCT GAT GCT TTA TCC CCA-3′); GAPDH (forward: 5′-GTC GGT GTG AAC GGA TTT G-3′; reverse: 5′-CTT GCC GTG GGT AGA GTC AT-3′). Each Ct value was normalized with GAPDH to determine relative expression, and experiments were carried out in triplicate. The data were analyzed with the 2-∆∆CT method.

***Western blot***

The protein was extracted using RIPA buffer, and the lysates were centrifuged to obtain supernatants to determine protein concentrations using a BCA protein assay kit. Then protein samples (50 μg) were loaded for electrophoresis on 10% SDS-PAGE, and the protein was transferred to a PVDF membrane (Millipore, Bedford, MA, USA). Then, the membrane was blocked with 5% skimmed milk in a TBS solution. The membrane was incubated overnight at 4 ^°^C against ANGPTL4 (1:400, ab196746, Rabbit polyclonal, Abcam, Cambridge, UK), PPARγ (1:500, sc-7273, Mouse monoclonal, Santa Cruz, USA), p-AKT (1:400, ab38449, Rabbit polyclonal, Abcam, Cambridge, UK), AKT (1:800, ab8805, Rabbit polyclonal, Abcam, Cambridge, UK), and GAPDH (1:2000, ab9485, Rabbit polyclonal, Abcam, Cambridge, UK) HRP-conjugated secondary antibodies (1:2000) were used to detect the immune reactivity of these target proteins. The protein band was visualized by ECL (Thermo, Waltham, MA, USA), and band density was determined using Image Software (Bio-Rad, Hercules, CA, USA).

***Statistical analysis***

Continuous variables are expressed as mean±SD. All data were analyzed by SPSS 19.0 statistical software. The comparison between the two groups was analyzed using a t-test. The three or more groups’ comparison was analyzed using one-way ANOVA, followed by Student-Newman-Keuls tests. The correlation between continuous variables was analyzed using Spearman’s rank correlation test. *P*<0.05 was considered a significant criterion for the difference.

## Results

***Serum ANGPTL4 was lower in AF patients and negatively correlated with indicators of atrial fibrosis***

**Figure 1:**
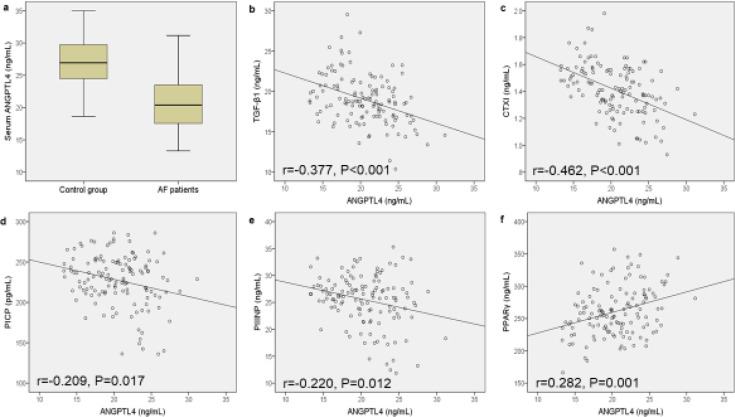
ANGPTL4 serum level is reduced in AF patients and negatively correlates with indicators of atrial fibrosis

We first measured ANGPTL4 serum concentration in the AF group and compared them with the control group, and the results showed that ANGPTL4 was significantly lower in the sera from the AF group than those from the control group (Figure 1A). In addition, we investigated the associations between serum ANGPTL4 and other serum indicators of atrial fibrosis. The results showed that ANGPTL4 level negatively correlated with the levels of TGF-β1 (Figure 1B), CTX-I (Figure 1C), PICP (Figure 1D), and PIIINP (Figure 1E) in sera from AF patients. Furthermore, serum ANGPTL4 positively correlated with the PPARγ level (Figure 1F). All these results indicate the potential role of ANGPTL4 in regulating atrial fibrosis.

***ANGPTL4 was down-regulated in Ang II-induced cardiac fibroblasts***

**Figure 2:**
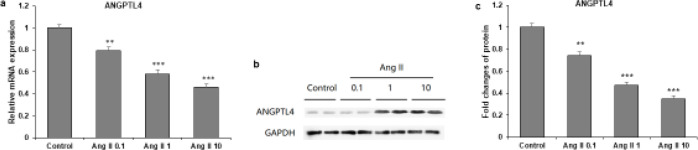
ANGPTL4 was down-regulated in Ang II-induced cardiac fibroblasts. Rat cardiac fibroblasts were incubated with Ang II at 0.1, 1, and 10 μM for 24 hr

To determine whether ANGPTL4 plays a role in atrial fibrosis, the ANGPTL4 expression level in Ang II-treated cardiac fibroblasts was measured by RT-qPCR and western blot. As shown in Figure 2A, ANGPTL4 mRNA was significantly down-regulated in cardiac fibroblasts after 24 hr Ang II treatment in a concentration-dependent manner (*P*<0.05). Moreover, compared with control cells, Ang II-treated cardiac fibroblasts exhibited lower expression of ANGPTL4 protein (Figure 2B-C). These data suggest the involvement of ANGPTL4 in atrial fibrosis.

***ANGPTL4 overexpression attenuated Ang IIinduced proliferation, migration, and collagen production of atrial fibroblasts***

**Figure 3.**
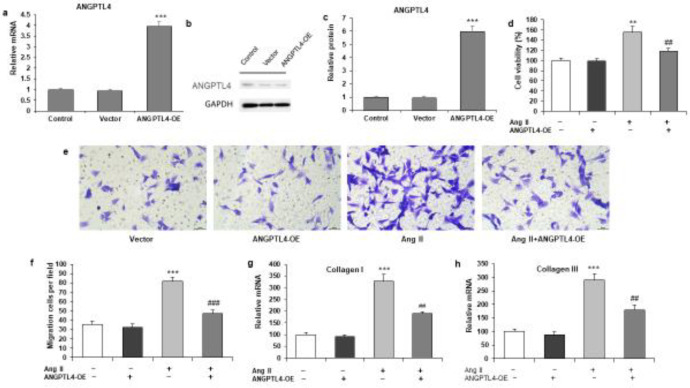
Overexpression of ANGPTL4 reduces Ang-II-induced proliferation, migration, and collagen production of rat cardiac atrial fibroblasts. ANGPTL4 overexpressing vectors (ANGPTL4-OE) and control vectors were transfected to atrial fibroblasts for 48 hr

Primary rat atrial fibroblasts with ANGPTL4 overexpression were constructed by transfecting cells with pcDNA3.1-ANGPTL4 vectors (ANGPTL4-OE) or empty vectors. After 48 hr, RT-qPCR and western blotting were carried out to confirm that the mRNA and protein levels of ANGPTL4 were effectively enhanced (both *P*<0001) (Figure 3A-C). To investigate the effects of ANGPTL4 overexpression on atrial fibrosis, we evaluated the impact of transfection of pcDNA3.1-ANGPTL4 on fibroblast proliferation, migration, and collagen production *in vitro*. We found that Ang II treatment caused a significant increase in fibroblast proliferation, and ANGPTL4 overexpression significantly suppressed cell proliferation according to the CCK8 assay (Figure 3D). The transwell migration assay results suggested that the ANGPTL4 overexpression suppressed the migration of atrial fibroblasts with Ang II stimulation (Figure 3E-F). However, ANGPTL4 overexpression did not influence the migration of atrial fibroblasts without Ang II. Moreover, ANGPTL4 overexpression attenuated the increase in collagen I and III mRNA levels after Ang II stimulation (Figure 3G-H). These results showed that Ang II-induced atrial fibroblast proliferation, migration, and collagen production were attenuated by ANGPTL4 overexpression.

***ANGPTL4 suppressed Ang II-induced atrial fibroblast proliferation, migration, and collagen production by targeting PPARγ***

**Figure 4:**
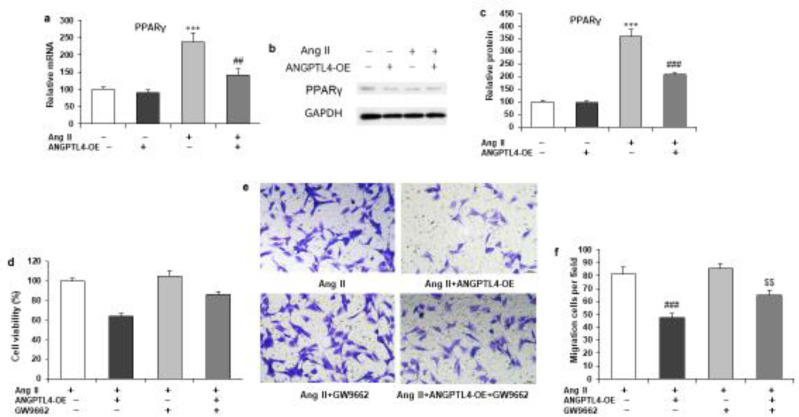
PPARγ is a target gene of ANGPTL4 in the proliferation, migration, and collagen production of rat cardiac atrial fibroblasts

To investigate the mechanisms of ANGPTL4 that attenuate the Ang II-induced proliferation, migration, and collagen production of atrial fibroblasts, we identified PPARγ as a potential target gene according to our previously published study. RT-qPCR and western blotting determined PPARγ mRNA and protein expression. Ang II strongly increased PPARγ gene expression in rat atrial fibroblasts, markedly reversed by ANGPTL4 overexpression (Figure 4A). Accordingly, the protein expression of PPARγ showed a similar change after ANGPTL4 overexpression (Figure 4B-C). Therefore, we used specific PPARγ inhibitor GW9662 (10 μM) to investigate the role of PPARγ in the effects of ANGPTL4 overexpression. The decrease in cell viability and migration by ANGPTL4 overexpression was partially reversed by co-incubation with GW9662 (Figure 4D-E). 

***ANGPTL4 activated PPARγ-Akt pathway in rat cardiac atrial fibroblasts***

**Figure 5:**
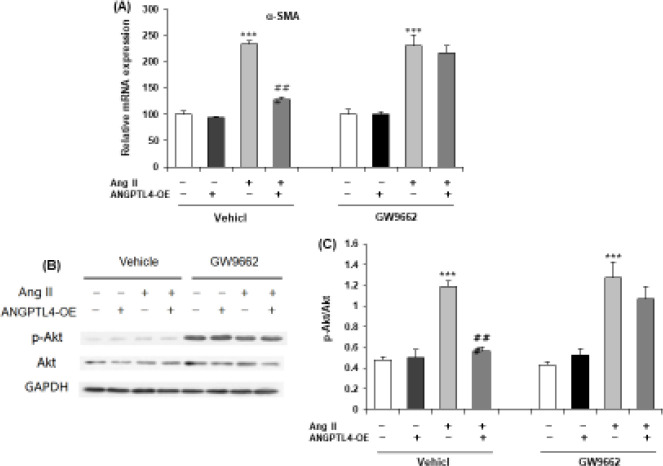
ANGPTL4 activates the PPARγ-Akt pathway in rat cardiac atrial fibroblasts

We then determined the α-SMA mRNA expression by RT-qPCR to investigate the transdifferentiation of atrial fibroblasts. ANGPTL4 overexpression significantly attenuated the Ang II-induced increase in α-SMA mRNA expression. Intriguingly, GW9662 alone did not affect the mRNA expression of α-SMA in fibroblasts with Ang II or ANGPTL4-OE but completely blocked the inhibitory effects of ANGPTL4 overexpression on fibroblasts transformation (Figure 5A). We also detected a downstream signal protein of PPARγ, Akt. Western blot showed ANGPTL4 overexpression decreased phosphorylation of Akt (p-Akt, T308) but did not change the expression of total Akt protein (Figure 5B). This effect was markedly diminished when fibroblasts were simultaneously incubated with GW9662 (Figure 5C).

A: ANGPTL4 concentration in serum from the AF group was compared with the control group by ELISA. Correlation of ANGPTL4 levels with serum levels of B: TGF-β1, C: CTX-I, D: PICP, E: PIIINP, and F: PPARγ. Data are representative of three experiments. ***P<0.001 vs control group

A: The expression of ANGPTL4 in rat cardiac fibroblasts was detected by RT-qPCR. B: Representative western blot bands of ANGPTL4 in rat cardiac fibroblasts. C: Quantification of the greyscale of protein bands of ANGPTL4. **P<0.01, ***P<0.001 vs control group

A: RT-qPCR was performed to assess mRNA levels of ANGPTL4. B: Western blotting was performed to assess ANGPTL4 protein levels, and representative images are shown. C: Bands were quantified by ImageJ software, and show the ANGPTL4 protein level is significantly increased after transfection. D: After transfection with ANGPTL4 or empty vector, fibroblasts were stimulated with 1 μM Ang II for 24 hr. Atrial fibroblast proliferation was evaluated in an MTT assay. E: Cell migration was measured via the Transwell migration assay. F: Statistical analysis of the number of migrated cells. G, H: mRNA levels of collagens I and III were evaluated by RT-qPCR. Data are representative of three experiments. **P<0.01, ***P<0.001 vs the vector group; ^##^*P<*0.01, ^###^*P<*0.001 vs Ang II group

A: Fibroblasts were transfected with ANGPTL4-OE or control vectors, followed by incubation with 1 μM Ang II for a further 24 hr. Quantification of PPARγ mRNA expression was performed by RT-qPCR. B: Representative western blot bands of PPARγ in rat cardiac fibroblasts. C: Quantification of the greyscale of protein bands of PPARγ. D: Fibroblasts were transfected with ANGPTL4-OE or control vectors, then treated with specific PPARγ inhibitor GW9662 (10 μM), followed by incubation with 1 μM Ang II for a further 24 hr. MTT assay was performed to determine cell viability. E: Cell migration was measured via Transwell migration assay. F: Quantification of the number of migrated cells. *P<0.05 vs the vector group; #*P<*0.05 vs Ang II group; $<0.05 vs Ang II+ANGPTL4-OE group

A: α-SMA mRNA expression was determined by RT-qPCR in atrial fibroblasts transfected with the ANGPTL4-OE, and incubated with GW9662 and Ang II. B: Representative western blot bands of Akt in rat cardiac fibroblasts. C: Alterations in the Akt pathway fibroblasts transfected with ANGPTL4-OE after GW9662 treatment. ***P<0.001 vs the vector group; ^###^*P<*0.001 vs Ang II group

## Discussion

In our study, we have investigated the potential protective effect of ANGPTL4 in Ang ll-induced AF. ANGPTL4 mRNA and protein expression were significantly decreased in Ang II-treated cardiac fibroblasts. Overexpression of ANGPTL4 potentially reduced Ang II-induced atrial fibroblast proliferation, migration, and collagen production in atrial tissue. The possible underlying mechanism could be associated with inhibiting different signaling proteins, such as PPARγ, α-SMA, and Akt. Thus, the study results demonstrated that ANGPTL4 could be a potential therapeutic target for treating AF.

Several studies showed that the down-regulation of ANGPTL4 is substantially correlated with lower levels of TG and a lower risk of cardiovascular events (19-21). ANGPTL-4 could be associated with lipid metabolisms (22, 23) and, taken together, suggested that ANGPTL-4 regulated TG in circulation by attenuating lipoprotein lipase and regulating free fatty acids uptake (24, 25). However, high-saturated fatty acid levels were observed in AF patients. They reduced the polyunsaturated fatty acid levels, which could be related to the increased inflammation, speculating that free fatty acid levels might play a critical role in the development and progression of AF (26). One research group reported that serum ANGPTL-4 concentrations potentially predict future cardiovascular disorders in patients with AF (19). In our study, we observed that ANGPTL4 was significantly reduced in patients with AF compared with the control group, speculating that serum ANGPTL4 could be a potential diagnostic and molecular biomarker.

The current study reported that ANGPTL4 was down-regulated in Ang II-induced cardiac fibroblasts. Therefore, Ang II is a crucial factor in AF and may stimulate atrial fibrosis. Thus, it increases the possibility of AF (16). The ANGPTL4 expression level was measured in Ang II-induced cardiac fibroblasts. The study results show lower expression of ANGPTL4 mRNA in Ang II-treated cardiac fibroblast cells after 24 hr in a concentration-dependent manner (Figure 2A). The results also demonstrate that ANGPTL4 protein expression was decreased in Ang II-treated cardiac fibroblast cells compared with control cells (Figure 2B-C). Our data speculate that ANGPTL4 is involved in atrial fibrosis.

We hypothesized that ANGPTL4 overexpression could decrease atrial fibroblasts’ cell proliferation, migration, and collagen production. We constructed ANGPTL4 overexpression (ANGPTL4-OE) using transfecting primary rat atrial fibroblasts cells with pcDNA3.1-ANGPTL4 vectors and confirmed ANGPTL4 mRNA and protein levels were potentially increased (Figure 3A-C). Next, we evaluated the possible effect of ANGPTL4 overexpression on atrial fibrosis consequences on fibroblast proliferation, migration, and collagen production *in vitro*. The results showed that Ang II treatment significantly increased fibroblast proliferation, but cell proliferation was potentially suppressed due to ANGPTL4 overexpression (Figure 3D). The same trends were observed in the cell migration assay. ANGPTL4 overexpression significantly inhibited atrial fibroblast migration with Ang II treatment (Figure 3E-F), but no influence was observed on the migration of atrial fibroblasts without Ang II. Moreover, it inhibited the increase in collagen I and III mRNA levels due to ANGPTL4 overexpression (Figure 3G-H). Accumulating evidence suggests that overexpression of ANGPTL4 potentially attenuated atrial fibroblast proliferation, migration, and collagen production with Ang II stimulation. 

 It is previously reported that reduced serum PPAR-γ was observed in elderly patients with AF (27), which suggests that it could be a potential target of AF. PPAR- γ activators may decrease the risk of AF by increasing atrial cell survival (28) and suppressing cardiac fibrosis and are involved in different risk factors for AF, including hypertension, diabetes, heart failure, and myocardial infarction (29). PPAR-α and PPAR-γ activate ANGPTL4 gene transcription and expression (30). A recent study showed that atrial PPAR-α tissue expression was lower in AF patients and might be correlated with atrial metabolic remodeling (31). In addition, ANGPTL4 also attenuates cardiac hypertrophy with increasing PPAR-α expression (32). Our results showed that ANGPTL4 overexpression potentially inhibited an increase in α-SMA mRNA expression with Ang II treatment. Interestingly, PPARγ inhibitor GW9662 did not influence the α-SMA mRNA expression in fibroblasts but completely attenuated the inhibitory effects of ANGPTL4 overexpression (Figure 5A). We also observed low protein expression of PPARγ, Akt. ANGPTL4 overexpression reduced the phosphorylation of Akt without affecting the total Akt protein expression (Figure 5B). However, this effect was significantly inhibited when atrial fibroblasts were simultaneously incubated with GW9662 (Figure 5C).

Our previous study found lower serum levels of ANGPTL4 in recurrent AF compared with new-onset AF (10). In contrast, our present study validated the expression of ANGPTL4 in Ang II-induced cardiac fibroblasts. The positive correlation between serum ANGPTL4 and PPARγ was revalidated in our manuscript. Moreover, we also found a positive correlation of ANGPTL4 with four other fibrotic biomarkers, TGF-β1, CTX-I, PICP, and PIIINP. Our results imply that ANGPTL4 might be associated with cardiac fibrosis. Our early study also shows ANGPTL4 attenuates Ang II-induced AF and atrial fibrosis in a mice model (18). The present study investigates the effect of ANGPTL4 overexpression (other than exogenous recombinant ANGPTL4 protein) on Ang II-induced cardiac fibroblasts. We found that PPARγ was activated by ANGPTL4 overexpression and that a PPARγ inhibitor, GW9662, can attenuate the profibrotic effect of ANGPTL4 overexpression. 

## Conclusion

The present study revealed that ANGPTL4 is a crucial factor in the regulation of AF, where it attenuated cell proliferation, migration, and collagen production in atrial tissue. Moreover, overexpression of ANGPTL4 inactivates the downstream PPARγ-Akt pathway in rat cardiac atrial fibroblasts. Therefore, increasing ANGPTL4 expression could be a potent strategy for AF treatment. This study provided a significant understanding of the regulation of AF and may provide a potential therapeutic target for the treatment of AF. However, the present study results still need to be further verified in a significant number of studies and clinical samples, a further mechanism study, and animal models.

## Authors’ Contributions

ZN designed the project, supervised the project, and revised the manuscript. XZ performed experiments and wrote the first draft of the manuscript. XZ, WG, and HZ helped perform the experiments and collect data. SH analyzed the data and performed statistical analysis.

## Conflicts of Interest

All authors declare that they do not have any conflicts of interest with other people or organizations.
